# A Case Report of Successful Management of Refractory Polyarticular Gout With Pegloticase

**DOI:** 10.7759/cureus.28390

**Published:** 2022-08-25

**Authors:** Dina Alnabwani, Ankita Prasad, Ashraf Raslan, Pramil Cheriyath

**Affiliations:** 1 Internal Medicine, Hackensack Meridian Ocean Medical Center, Brick, USA; 2 Internal Medicine, Ocean University Medical Center, Brick, USA; 3 Rheumatology, Valley Health System, Ridgewood, USA; 4 Internal Medicine, Hackensack Meridian Health Ocean Medical Center, Brick, USA

**Keywords:** tophi, methotrexate, inflammatory polyarthritis, msu crystal load, pegloticase, dual energy computed tomography, seronegative rheumatoid arthritis, gouty arthritis, gout

## Abstract

Gout is inflammatory arthritis and is easily recognizable by healthcare providers by its typical clinical presentation of acute gout flare or by the presence of chronic tophaceous deposits. However, chronic gouty arthropathy can be more challenging to diagnose in some cases, especially in the absence of a previous history of gout and other characteristic findings on exam. We present a case of chronic gouty arthropathy with features mimicking rheumatoid arthritis involving multiple small joints of hands and feet and other large joints. He had high serum uric acids and a dual-energy CT (DECT) scan of the feet and ankles was obtained which showed polyarticular gout. He was started on pegloticase in view of joint erosions, and severe limitations in activity which resulted in a lowering of monosodium urate crystals and symptomatic improvement.

## Introduction

Gout is inflammatory arthritis, affecting about 3% of the adult population in the United States [1]. In most cases, it is easily recognizable by healthcare providers by its typical presentation of an acute gout flare or by the presence of chronic tophaceous deposits. However, chronic gouty arthropathy can be more challenging to diagnose in some cases, especially in the absence of a previous history of gout and other characteristic findings on exam. We present a case of chronic gouty arthropathy with features mimicking seronegative arthritis. Dual-energy CT (DECT) was instrumental in diagnosing, but his uric acid levels did not improve on allopurinol and febuxostat. Pegloticase helped reduce uric acid levels and gave symptomatic relief and radiological improvement.

## Case presentation

**Figure 1:**
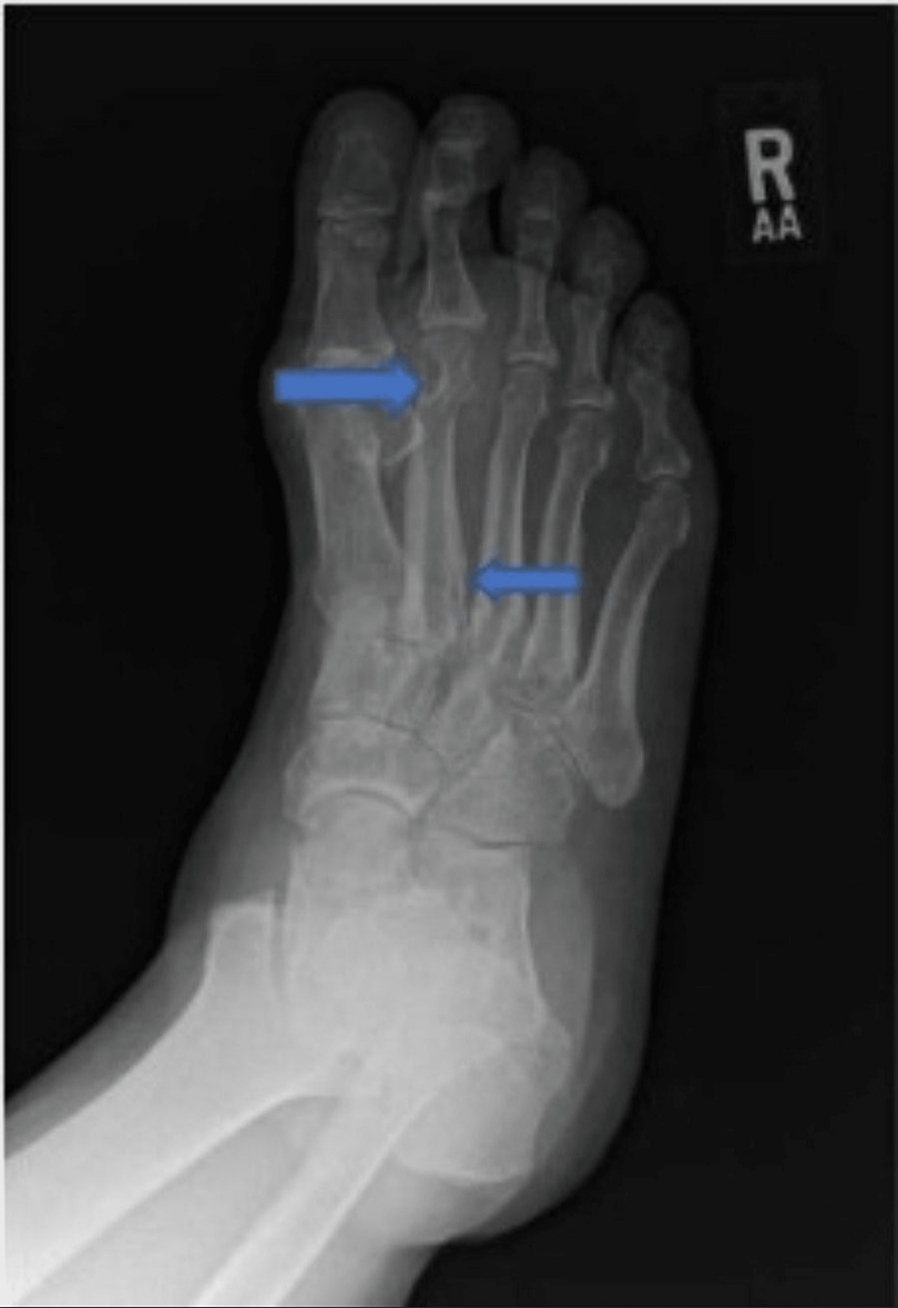
X-ray right foot showing periarticular osteopenia and erosions of the metatarsophalangeal and interphalangeal joints. Blue arrows point to the erosions, and generalized osteopenia is seen in the image.

Our patient is a 62-year-old male with a history of hypertension who initially presented to our rheumatology office in November 2019 for the evaluation of chronic joint pain. His joint pain started in early 2017 and mainly affected his hands, knees, ankles, and feet. He described the pain as constant, sharp, severe at times, and worse in the morning and with activity. The severity of his foot and ankle pain caused difficulty in walking. His right hand always felt stiff, and he had morning stiffness in other joints for about 30 minutes. His exam noted tenderness in his right second metacarpophalangeal joints and bilateral metatarsophalangeal joints. He had a dry skin patch on the dorsum of his right foot and onycholysis of the right first and second toenails. He had no joint swelling, no synovitis, and the rest of the musculoskeletal examination was unremarkable. Laboratory testing revealed a positive antinuclear antibody (ANA) 1:160 dense speckled pattern, negative rheumatoid factor (RF), negative anti-cyclic citrullinated peptide (CCP)** **and negative etaprotein, normal erythrocyte sedimentation rate (ESR)and C-reactive protein (CRP), and uric acid of 11.4 mg/dl (3.5 - 7.2 (mg/dL). X-rays of the feet showed periarticular osteopenia and periarticular erosions of the metatarsophalangeal and interphalangeal joints (Figure 1), described by the radiologist as most consistent with rheumatoid arthritis (RA).

**Figure 2:**
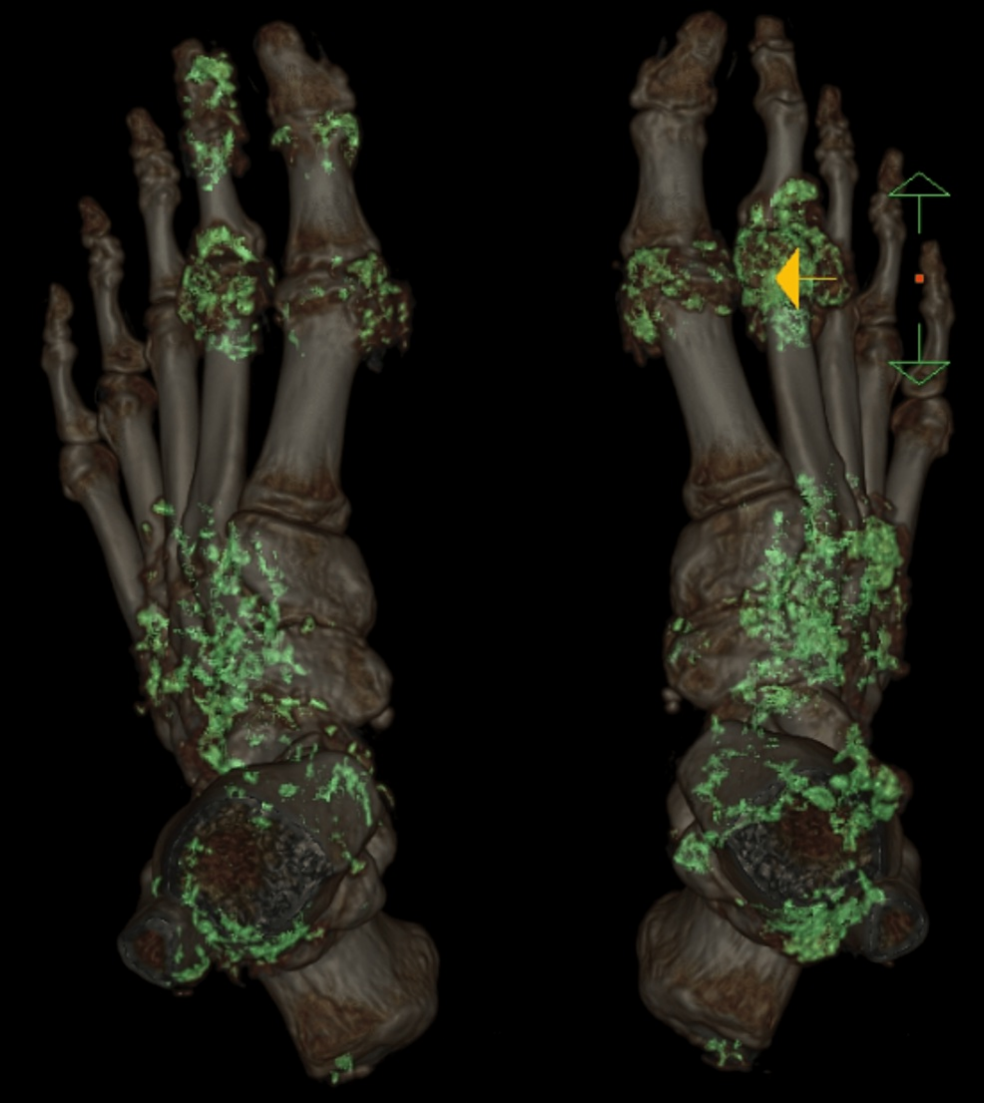
DECT images of feet showing heavy MSU crystal deposition DECT: dual energy CT; MSU: monosodium urate

**Figure 3:**
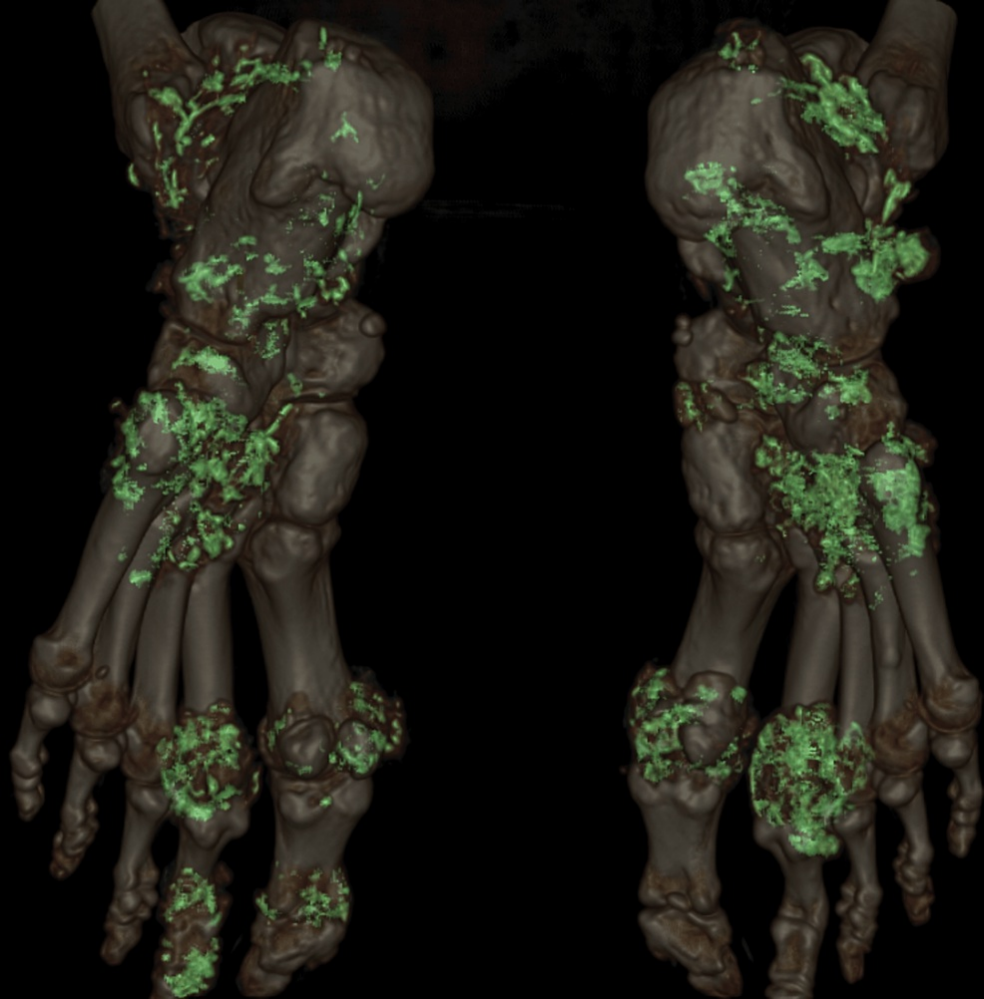
DECT images showing extensive MSU deposition in feet. DECT: dual energy CT; MSU: monosodium urate

Seronegative RA was suspected at this point. With a markedly elevated uric acid level, a DECT scan of the feet and ankles was obtained to rule out polyarticular gout. It showed extensive monosodium urate crystal deposition in the feet and ankles, with changes consistent with advanced gouty arthritis (Figures 2-3)

**Figure 4:**
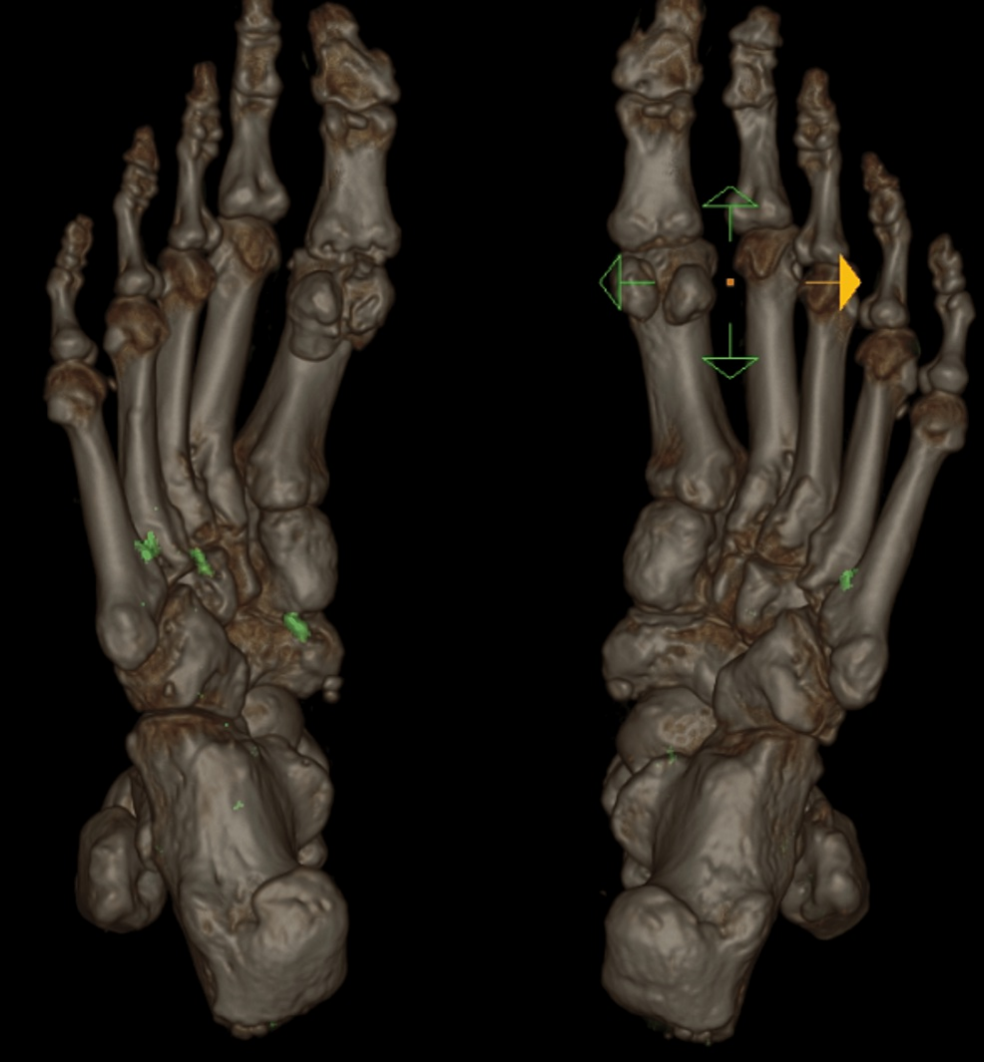
DECT images of both feet showing resolution of MSU deposition after treatment. DECT: dual energy CT; MSU: monosodium urate

The patient was diagnosed with advanced polyarticular gout. Given the severity of his gouty arthritis with severe pain affecting mobility and the use of his hands for work and daily life activities, the presence of erosions and advanced gouty arthritis changes on imaging, and evidence of high monosodium urate (MSU) crystal burden, the decision was made to start pegloticase (PEG) therapy. However, we could not begin this therapy due to the high cost and inability to obtain insurance approval for the drug. He was started on allopurinol, and the dose was titrated up to 700 mg daily, but the uric acid level remained above 7.5 mg/dl. His target serum uric acid level was less than 5 mg/dl. Allopurinol was stopped, and the patient was started on febuxostat, which was titrated up to 80 mg/d but failed to achieve the target uric acid level. His symptoms continued to worsen gradually. We planned to add probenecid. At this point, we were able to obtain insurance approval for PEG therapy. Febuxostat was discontinued and PEG was started in January 2021. His uric acid level became undetectable after the first infusion (uric acid <0.2 mg/dl) and remained undetectable throughout the following 11 months of therapy. He was started on methotrexate at 15 mg once weekly before starting PEG therapy to decrease the likelihood of developing anti-pegloticase antibodies. He was also on colchicine at 0.6 mg/d and prednisone at 2.5 mg/d. His joint pain and stiffness improved significantly within 2-3 months of starting pegloticase therapy. A repeat DECT scan of the feet and ankles on 11/2021 showed a marked decrease of the MSU crystal deposition with persistent erosive changes similar to the previous study (Figure 4).

PEG therapy was discontinued in December 2021 and the patient was successfully transitioned to allopurinol.

## Discussion

Gout can be easily recognized due to its characteristic findings in acute gout flares or chronic tophaceous disease. However, unusual cases of chronic gouty arthritis can be challenging to diagnose because most healthcare providers associate gout with its characteristic symptoms of acute flares and tophi. Gout can get easily overlooked if these symptoms are not present and misdiagnosed as another form of inflammatory polyarthritis like seronegative rheumatoid arthritis [2]. Our case represents one of these challenging cases where the patient did not have a prior history of gout or typical gout flares and had no apparent tophaceous deposition present on exam. He presented with chronic symmetrical pain and stiffness, mimicking rheumatoid arthritis. In addition, his plain X-rays showed marginal erosions that the radiologist described as consistent with rheumatoid arthritis.

The case highlights the utility of the DECT scan in establishing the diagnosis of gout in such complex cases [3]. The DECT scan was also helpful in assessing the MSU crystal burden and hence helped guide our therapeutic plan. PEG was an effective and well-tolerated therapy in refractory gout in our patient and helped rapidly decrease the disabling arthritis symptoms of the patient and dramatically improve the MSU crystal burden, as evidenced by follow-up DECT scan imaging of his feet and ankles. Concomitant immunosuppressive therapy with PEG is essential to decrease the likelihood of developing anti-pegloticase antibodies and is usually well-tolerated [4]. The studied immunosuppressive therapies include methotrexate, azathioprine, and mycophenolate mofetil [5]. In our patient, we successfully used methotrexate (15 mg) once weekly, which was started before initiating PEG and maintained throughout the treatment course.

## Conclusions

Polyarticular gout should always be considered in the differential diagnosis of seronegative RA. Dual-energy CT scanning can be essential in establishing or ruling out gout diagnosis and assessing the MSU crystal burden. PEG is an effective therapy for refractory polyarticular gout. Unless contraindicated, it should be used concomitantly with immunosuppressive therapy (methotrexate in our case) to decrease the likelihood of developing anti-pegloticase antibodies.
